# Structural Basis for Mutations of Human Aquaporins Associated to Genetic Diseases

**DOI:** 10.3390/ijms19061577

**Published:** 2018-05-25

**Authors:** Luisa Calvanese, Gabriella D’Auria, Anna Vangone, Lucia Falcigno, Romina Oliva

**Affiliations:** 1 CIRPeB, University of Naples Federico II, Napoli I-80134, Italy; luisa.calvanese@unina.it (L.C.); gabriella.dauria@unina.it (G.D.); falcigno@unina.it (L.F.); 2 Department of Pharmacy, University of Naples Federico II, Napoli I-80134, Italy; 3 Institute of Biostructures and Bioimaging, CNR, Napoli I-80134, Italy; 4 Bijvoet Center for Biomolecular Research, Faculty of Science, Department of Chemistry, Utrecht University, Padualaan 8, 3584 CH Utrecht, The Netherlands; a.vangone@gmail.com; 5 Department of Sciences and Technologies, University Parthenope of Naples, Napoli I-80143, Italy

**Keywords:** Aquaporins, mutations, genetic diseases, SAPs, structural effect, membrane proteins, channel proteins, NDI, PPKB

## Abstract

Aquaporins (AQPs) are among the best structural-characterized membrane proteins, fulfilling the role of allowing water flux across cellular membranes. Thus far, 34 single amino acid polymorphisms have been reported in HUMSAVAR for human aquaporins as disease-related. They affect AQP2, AQP5 and AQP8, where they are associated with nephrogenic diabetes insipidus, keratoderma and colorectal cancer, respectively. For half of these mutations, although they are mostly experimentally characterized in their dysfunctional phenotypes, a structural characterization at a molecular level is still missing. In this work, we focus on such mutations and discuss what the structural defects are that they appear to cause. To achieve this aim, we built a 3D molecular model for each mutant and explored the effect of the mutation on all of their structural features. Based on these analyses, we could collect the structural defects of all the pathogenic mutations (here or previously analysed) under few main categories, that we found to nicely correlate with the experimental phenotypes reported for several of the analysed mutants. Some of the structural analyses we present here provide a rationale for previously experimentally observed phenotypes. Furthermore, our comprehensive overview can be used as a reference frame for the interpretation, on a structural basis, of defective phenotypes of other aquaporin pathogenic mutants.

## 1. Introduction

Aquaporins (AQPs) are highly selective transmembrane channel proteins, which allow flux of water and small solutes across biological membranes in a large variety of organisms [1]. Orthodox aquaporins are selective for water, while aquaglyceroporins are additionally permeable to glycerol and other small, polar solutes [1]. In humans, 13 AQPs have been identified. Seven of them, AQP0, AQP1, AQP2, AQP4, AQP5, AQP6 and AQP8, are orthodox aquaporins; however AQP0 is known to show a narrower pore and a lower permeation efficiency [2,3,4], while AQP8 is additionally permeable to ammonia and different in phylogeny from other orthodox AQPs [5,6,7,8]. Four human AQPs, AQP3, AQP7, AQP9 and AQP10, are instead classified as aquaglyceroporins, being permeable to glycerol and other small solutes such as urea and/or ammonia [9]. Finally, human AQP11 and AQP12 are hardly classifiable as either classical aquaporins or aquaglyceroporins. They have indeed been proposed as a third group, the superaquaporins [10] or subcellular aquaporins, according to their cellular localisation [11], although they seem to preserve the structural arrangement of other aquaporins [12].

Nowadays, numerous experimental structures are available for aquaporins [13]. Among these, 10 structures have been solved for 4 human AQPs, AQP1, AQP2, AQP4 and AQP5, at resolutions better than 3.8 Å (see Table 1). The availability of experimentally-determined AQP structures over different organisms and subfamilies has allowed the determination of some conserved structural features: Six trans-membrane helices surround a narrow water-conducting channel, while two cytoplasmic loops dip into the membrane from opposite sides, forming two shorter helices that together constitute a seventh pseudo-trans-membrane segment. In the membrane, four aquaporin molecules are assembled to form a homotetramer. Each monomer exhibits two conserved Asn-Pro-Ala (NPA) sequence motifs, which cap the end of the two shorter helices and lie in the middle of the permeation channel, forming a constriction. Another constriction, known as the aromatic/Arg (ar/R) selectivity filter, is located at the extracellular side of the channel and is formed by four residues, including a highly conserved arginine and an aromatic residue, such as phenylalanine or tryptophan. The ar/R filter is the most significant and conserved difference between the aquaporin and aquaglyceroporin channels, in terms of sequence and consequently structure and selectivity [14,15].

AQPs also have intracellular amino (N-) and carboxyl (C-) termini of variable length. For instance, AQP11 has a large N-terminal tail of ~40 residues, whereas AQPs 1, 2 and 5 have short N-termini of less than 10 residues. The C-terminal tail is only ~10 residues long in AQP8, while it is 45 and 40 residues long in AQP2 and AQP5, respectively. The majority of AQP interaction partners bind to their C-terminus, which frequently hosts post-translational modifications sites, in particular phosphorylation sites, as possible modulators of the interactions with the regulatory proteins. This allows for a dynamic control of the regulatory process [16]. Due to the difficulties of obtaining well-diffracting crystals of the full-length AQPs, in most available AQP experimental structures the protein constructs are truncated and do not include the N- and C-terminal tails. This implies a limited structural knowledge of the AQP terminal regions and suggests their intrinsic flexibility [5].

The mammalian aquaporins are expressed in a tissue-dependent manner and serve a diverse range of physiological functions, such as maintenance of eye lens transparency, urine concentration in the kidney, skin hydration in epidermal keratinocytes and the secretion of tears, saliva and sweat [17,18]. Dysfunctional aquaporins have been linked to a number of pathological conditions, including brain oedema, renal disease, obesity and cancer, raising their attractiveness as drug targets [13,18].

In HUMSAVAR (from the Uniprot/Swiss-Prot protein knowledgebase, [19]), thirty-four non-synonymous single amino acid polymorphisms (SAPs) are reported for human aquaporins, which are associated with genetic disorders, such as nephrogenic diabetes insipidus (NDI), keratoderma, and breast cancer. Most of the above SAPs occur in AQP2, an aquaporin found in the kidney collecting duct, where it trans-locates water across the apical membrane and is crucial for urine concentration [20]. AQP2 is regulated by trafficking between intracellular storage vesicles and the apical membrane, a process that is tightly controlled by the pituitary hormone arginine vasopressin. Defective AQP2 trafficking causes NDI, a water balance disorder characterized by large urine volumes, leading to dehydration. Although congenital NDI is most commonly caused by mutations in the gene for the V2 vasopressin receptor, around 10% of NDI patients harbor AQP2 gene mutations [21]. NDI-causing AQP2 mutations either interfere with its shuttling from storage vesicles to the apical membrane or, more frequently, cause misfolding and retention in the endoplasmic reticulum (ER) [22,23].

A few other SAPs are instead located on AQP5, a cell-membrane protein that allows osmotic movement of water across the cell membrane independently of solute transport [24]. Aquaporin 5 is situated in the apical plasma membrane of cells of the sweat glands, salivary glands, lacrimal gland, lung, cornea [24] and in the palmoplantar epidermis [25]. An autosomal dominant form of diffuse non-epidermolytic palmoplantar keratoderma, a palmoplantar keratoderma of the Bothnian type (PPKB), is caused by mutations on AQP5, leading to defective epidermal-water-barrier function in the epidermis of the palms and soles [26]. Finally, a disease-related SAP is located on AQP8. Aquaporin 8 has been detected in many tissues and organs [27,28,29]. It has been hypothesised to play a role in spermatogenesis, fertilisation, and the secretion of pancreatic juice and saliva [27,30,31,32]. The expression level of AQP8 in colonic epithelial cells has been shown to be related with colorectal tumors [33].

For half of these mutations, an insight of the defective phenotypes on a structural basis is reported in References [21,25]. For the remaining mutations, although mostly experimentally characterised in their dysfunctional phenotypes, a structural characterization at a molecular level is still missing. In this work, we focus on such mutations and discuss what seem to be their structural defects. To this aim, we built a molecular model for each mutant and explored the effect of the mutation on: the pore dimension and amino acid conservation, and the intra-/inter-monomer helices packing. The surface electrostatic potential was also calculated when relevant. Based on these analyses, we could classify the structural defects of all the pathogenic mutations (those analysed here and those analysed previously) under a few main categories, i.e., mutations affecting: (i) the pore features, (ii) the tetramer assembly, (iii) the monomer folding, and (iv) the C-terminal tail signal for post-translational phosphorylation*.* Furthermore, we found these categories to nicely correlate with the experimental phenotypes reported for several of the analysed mutants. Therefore, we believe this study can represent a reference frame for both a deeper understanding of the structure-function relationship in the AQP family and the possible prediction, on a structural basis, of the defective phenotypes of other AQP mutants.

## 2. Results and Discussion

Thirty-four SAPs in aquaporins are reported in the Uniprot/Swiss-Prot protein knowledgebase (see Table 2). Twenty-eight of them affect AQP2 and are associated with NDI, 5 affect AQP5 and are associated to PPKB, while 1 affects AQP8 and is associated with colorectal tumor, although its type of variant is reported as ‘unclassified’. A functional characterisation is available for most of these mutations. In addition, for 12 AQP2 and the 5 AQP5 mutants the structural basis for the defective phenotypes has been previously discussed [21,25].

**Figure 1:**
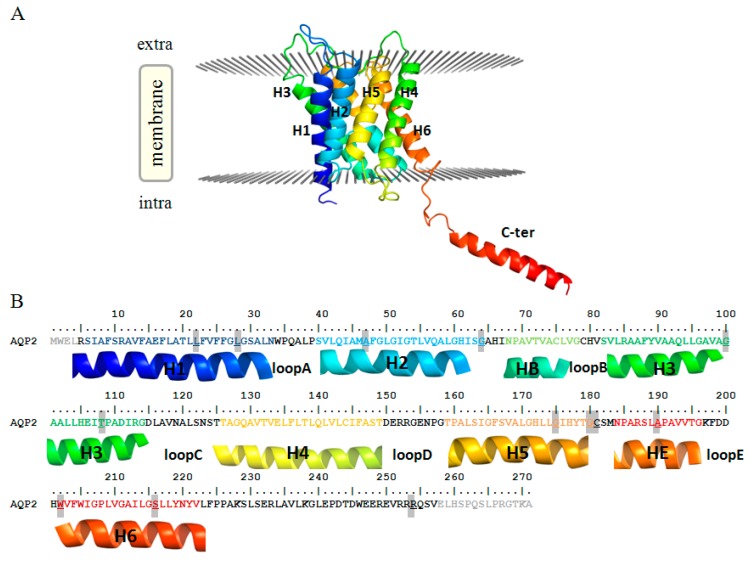
Human AQP2 monomer (**A**) structure (PDB ID: 4OJ2) and (**B**) sequence. In both panels, each helix is identified with the same colour and label. Mutated residues here examined are highlighted in the sequence (**B**). The N- and C-terminal residues not present in the PDB X-ray structure are shown in light gray.

Herein, we focused on the remaining 17 mutations. Indeed, while a detailed functional characterisation is available for most of them, a rationale of the exhibited phenotypes on a molecular basis is still missing. In particular, for 15 mutants (14 for AQP2 and 1 for AQP8, highlighted in Table 2) we built molecular models and performed several structural analyses to assess the structural flaws possibly associated with their dysfunctional phenotypes. To facilitate the discussion about the position and structural impact of such mutations, both structures and sequences of human AQP2 and AQP8 have been reported in Figure 1 and Figure S1, respectively.

Of the 14 modeled AQP2 mutations, 12 cause autosomal recessive NDI and are located in the trans-membrane region of the protein, spreading between the first (Helix-1) and the last (Helix-6) trans-membrane helix. Two more AQP2 mutations, both involving the sequence position 254 (R254L, R254Q), cause instead autosomal dominant NDI and are located on the protein C-terminal tail.

In the following account, we report the structural analysis and discussion of such mutations, all of them highlighted in Table 2. We classified them into four main groups according to the molecular irregularity that each can cause. We identified mutations affecting: (1) the pore, (2) the tetramer assembly, (3) the monomer folding, and (4) signal loss for the protein phosphorylation.

Finally, in order to provide a complete picture of AQP mutations and their structural effect, we briefly list the structural insights previously reported in literature regarding the other pathogenic mutants (reported in Table 2 and not highlighted). For 2 AQP2 mutations, E258K and P262L, lacking a structural characterisation, as they belong to a region of the AQP2 C-terminal tail not present in the available experimental structures, available functional data are concisely recalled.

### 2.1. Mutations Affecting the Pore (AQP2-G64R, AQP2-G180S)

**Figure 2:**
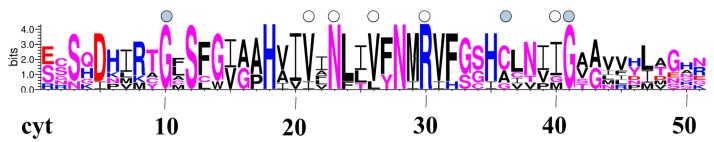
Pore logo representation of human AQPs. Sequence logo of the pore-lining residues for the seven human orthodox AQPs. The logo numbers correspond to the structural position of the residues along the channel axis and not to their sequence position. Each pore is orientated from the intracellular (left) to the extracellular (right) side. Acidic residues are coloured in red, basic residues in blue, hydrophobic residues in black and polar residues in purple. The pore positions where mutations occur, which are hypothesized to affect the pore features herein or in previous studies [21,25], are pointed out by a filled or an empty circle, respectively.

Two mutations, G64R and G180S, involve two glycines lining the pore of the AQP2 monomer. In Figure 2 we report a pore-logo representation derived for human aquaporins. This shows the sequence conservation of the 51 residues lining the pore, from the cytoplasmic (left) to the extracellular (right) side. In this representation, amino acids are ordered geometrically along the channel axis, i.e., adjacent amino acids are not necessarily adjacent in the sequence, but they lie adjacent in the channel, giving an immediate view of the composition of the pore. We have proposed and used previously this representation for the comparison of aquaporin pores from different species/subfamilies [12,45,46,47]. In the pore-logo, G64 and G180 occupy the pore positions 10 and 41, on the cytoplasmic and extracellular side of the channel, respectively. They are fully conserved among all human canonical and glycerol-permeable AQPs (see also Figure S2).

**Figure 3:**
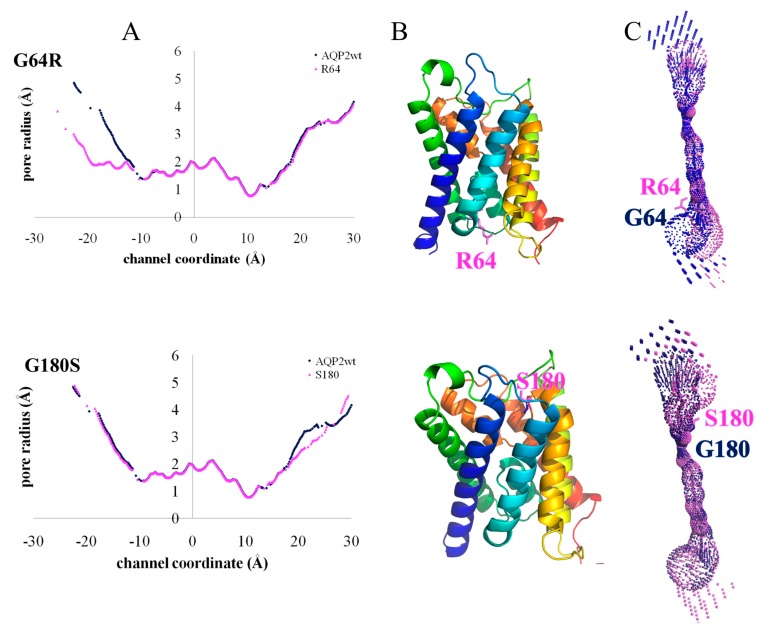
Mutations causing pore narrowing. (**A**) Pore radius along the channel axis for AQP2 wild type (blue) and mutants (violet); (**B**) cartoon representation of mutant models with mutated residues in violet sticks; (**C**) pore surface dimensions of AQP2 wild type (blue spheres) and mutants (violet spheres) calculated with HOLE [49].

Due to their position, the steric hindrance caused by the mutations involve the pore region, seeming to interfere with the pore shape and dimension rather than the monomer folding. Therefore, we calculated the pore radius along the channel of both the G64R and G180S mutants and compared it with the wt-AQP2 values (see Figure 3). From Figure 3, it is clear that the G64R mutation significantly reduces the pore radius of the protein at the cytoplasmic side (at 10–20 Å from the pore center along the channel axis, Figure 3A), while the G180S mutation induces a smaller reduction of the pore radius at the extracellular side (≈20 Å from the pore center, Figure 3A) just above the ar/R constriction site.

Our structural analysis is in agreement with experimental studies available for G64R (no functional characterization is instead available for G180S), showing that the mutation results in a functional protein, retaining approximately 20% of the single-channel water permeability of the wt-AQP2 [48]. This finding was considered surprising by the authors of the experimental study, since such a mutation changes the hydrophobic G64 into the charged arginine and affects a highly conserved residue. Our analysis provides a rationale for this, showing that the mutation does not interfere with the correct folding of the monomer; it just narrows it, thus causing a significant reduction in the permeation efficacy as experimentally observed.

### 2.2. Mutations Affecting the Tetramer Assembly (AQP2-L22V)

**Figure 4:**
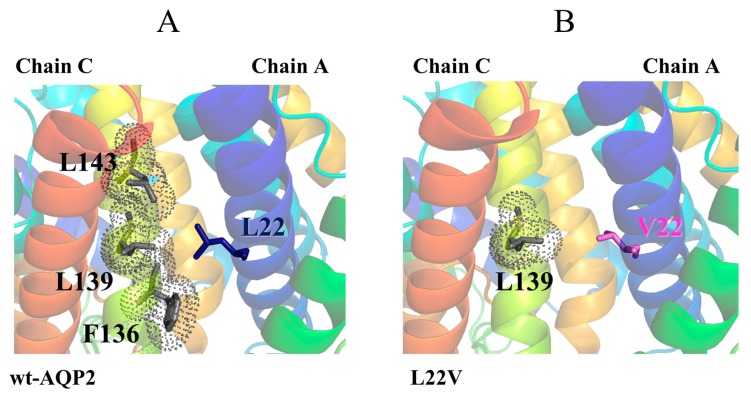
Tetramer assembly affecting L22V mutant. Cartoon representation of: (**A**) wt-AQP2 and (**B**) L22V mutant with discussed residues shown as sticks and labeled. The interacting residues are shown as gray Connolly surface.

Leucine 22 is located in the middle of AQP2 Helix-1, at the inter-monomer interface, where it participates in a hydrophobic patch with several residues, such as the F136, L139 and L143 on Helix-4 of the adjacent monomer (Figure 4). Mutation of that leucine to valine, with the consequent shortening of the hydrophobic side chain, causes the loss of interactions with residues L139 and F136. Therefore, the mutation is expected to have no effect on the structure and functionality of the single monomers, but rather to affect the packing interactions between the monomers.

This is in agreement with experimental studies showing mild phenotypes for this mutant. The single-channel water permeability of the L22V mutant is indeed similar to that of the wild-type protein (osmotic water permeability, Pf, is ∼60% of that for wt-AQP2 [50] when expressed in *Xenopus oocytes* [51]). In addition, in pulse-chase experiments in transfected Chinese hamster ovary (CHO) cells, the rate of degradation of L22V is comparable to that of the wild-type [51], confirming the mutant stability. Finally, only a partial accumulation in the endoplasmic reticulum (ER) was observed for this mutant, with transiently transfected cells expressing L22V showing a mixed staining pattern between the wild-type protein (plasma membrane) and a mutant with compromised folding (ER accumulation) [50].

### 2.3. Mutations Affecting the Monomer Folding (AQP2-L28P, AQP2-A47V, APQ2-G100V, AQP2-T108M, AQP2-G175R, AQP2-C181W, AQP2-A190T, AQP2-W202C, AQP2-S216P, AQP8-I229M)

**Figure 5:**
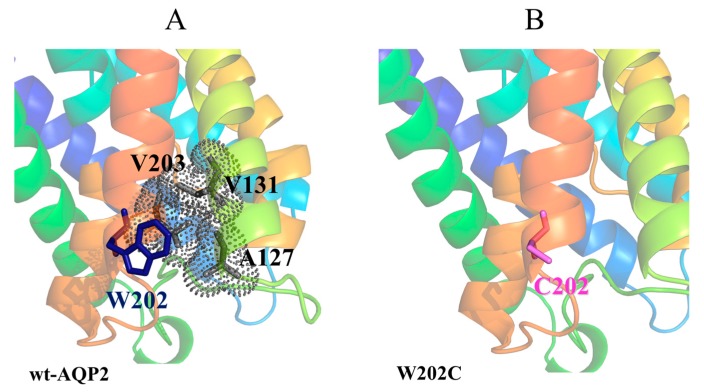
Structural representation of W202C mutant. Cartoon representation of: (**A**) human AQP2 wild type and (**B**) W202C mutant with native and mutated residues shown as blue and violet sticks, respectively. The residues interacting with W202 in AQP2 wild type are shown as gray sticks with dot surface, whilst in W202C mutant no interactions involving C202 are found. The discussed residues are labeled.

**Figure 6:**
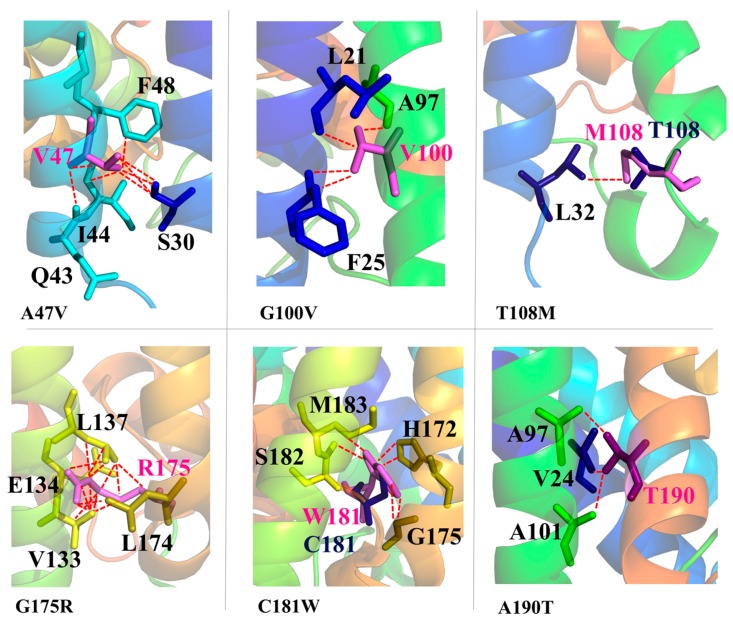
Mutations causing intra-monomer clashes. Cartoon representation of AQP2 mutants with mutated residues shown as violet sticks and clashing residues colored as the helix to which they belong. Dotted red lines indicate clashes. The wild type residues are shown in blue only when different from Gly or Ala, i.e., for T108 and C181, to pinpoint the lack of clashes in these cases.

**Figure 7:**
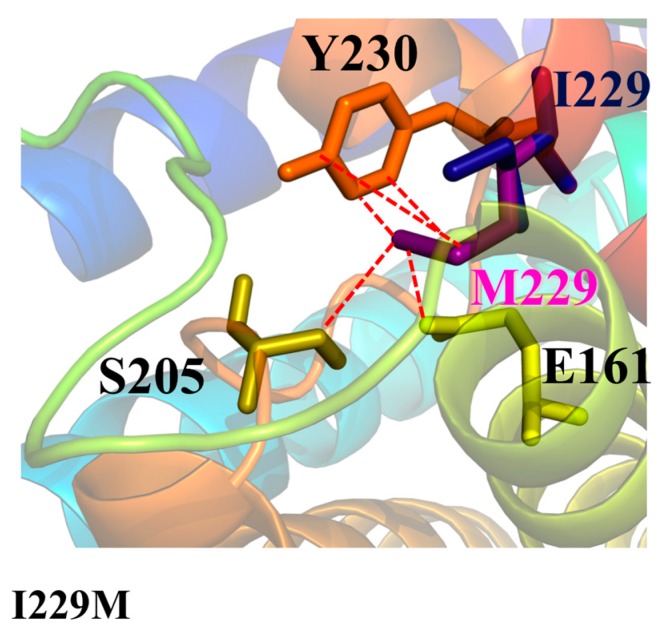
Cartoon representation of human AQP8 and its mutant I229M with discussed residues shown as sticks. Clashes between residues are shown in red dashed lines.

Nine AQP2 mutations plus the AQP8-I229M mutation seem to impair the correct monomer folding (Table 2 and Figure 5, Figure 6 and Figure 7). Two of the AQP2 mutants, L28P and S216P, introduce a proline residue, well-known to destabilize α-helices (psi dihedral angles restricted in the range of 145–160 degrees) in the middle of Helix-1 and Helix-6, respectively. The S216P and L28P mutants have been indeed proved to be unstable, non-functional aquaporins, with a water transport rate when expressed in oocytes not different from the controls [48,52]. Such data are in agreement with the rationale we propose: an incorrect folding of the monomer, caused by helices destabilization, would indeed affect the function of the aquaporin, as observed.

The mutation W202C, on Helix-6, also destabilises the monomer, but it does this by loosening the network of interactions within intra-monomer helices, as shown in Figure 5. In particular, while the wild-type W202 residue is involved in hydrophobic interactions with A127 and V131 on Helix-4, and with V203 on Helix-6, such interactions are missing in the W202C mutant.

Finally, other six AQP2 mutations, A47V, G100V, T108M, G175R, C181W and A190T, introduce steric clashes with surrounding residues (Figure 6). In all these cases, the mutated residue indeed adds a steric hindrance at the interface with other structural motifs of the monomer.

In particular, the A47V mutation on Helix-2 introduces steric clashes with the neighbor I44 and F48 on the same helix, and with S30 on Helix-1; G100V and T108M, on Helix-3, affect the packing with Helix-1, causing clashes with L21 and F25 and with L32, respectively; G175R, on Helix-5, causes clashes with V133, E134, L137 on Helix-4; A190T, on Helix-E, affects the packing with Helix-3, giving clashes with residues A97 and A101. C181W represents a special case, as C181 is located at the beginning of the unstructured intra-membrane region between Helix-5 and -6, where it lines the pore (pore-logo position 36, Figure 2). It also represents a site of inhibition of water permeation by mercury compounds [53,54]. It has been shown that the C181W mutation results in a non-functional water channel [51]. As we show here, the C181 substitution to tryptophan indeed introduces such a large structural strain (clashes with residues H172, G175 and M183) to interfere with the proper monomer folding, rather than just narrowing the pore itself. Overall, our structural analysis suggests the above mutants to be non-functional due to impaired functional folding.

For all the above six mutations, indeed, expression in oocytes resulted in a water permeability not significantly different from non-injected oocytes, except for A47V, which was shown to retain approximately 40% of the single channel water permeability of wt-AQP2 [50,52,55,56,57,58,59]. In addition, pulse-chase experiments in transfected CHO cells showed that the rate of degradation of C181W is accelerated when compared to wt-AQP2 [51] and other studies have reported that A190T, C181W and A47V are misfolded and retained in the ER [50,52,56,60], as found for other AQP2 mutants causing recessive NDI [22,24], while retention of G175R in the ER is debated [55]. Such experimental data are all in agreement with a non-functional structure of the mutants as we propose.

An analogous structural effect is observed for the only AQP8 mutant, I229M. I229, on Helix-6, is a buried hydrophobic residue in the wt-AQP8 monomers, involved in several hydrophobic interactions (Figure 7). Its mutation to a bulkier methionine necessarily introduces steric clashes with neighbor residues. These clashes can disturb the correct folding of the AQP8 monomer. Therefore, although a functional characterisation is not yet available for validation, this mutant is expected to feature severe phenotypes similar to those observed for the AQP2 mutants with an analogous structural defect, i.e., to be a non-functional aquaporin, with likely ER retention. A possible misfolding for such mutant has also been proposed in Reference [61].

### 2.4. Mutations Affecting the Protein Phosphorylation (AQP2-R254L, AQP2-R254Q)

As mentioned in the introduction, post-translational modifications in the AQP C-tail can control the trafficking processing by acting as signals for the cellular trafficking system. The two mutations on the AQP2 C-tail, R254L and R254Q, lead to the loss of a salt bridge that R254 would normally form with E250 (Figure S3). However, this is not expected to affect the stability of the protein structure. Instead, it affects the network of charged residues (which are highly present in this protein region) and, possibly, the phosphorylation of S256, shown to have crucial importance in the AQP2 translocation to the apical membrane [62], 2 residues downstream.

The lack of arginine vasopressin-mediated phosphorylation of AQP2 at S256 has been shown to be the sole cause of dominant NDI for the R254L and R254Q mutations. When expressed in oocytes, indeed, AQP2-R254L and R254Q appear to be functional water channels but impaired in their transport to the cell surface to the same degree as AQP2-S256A, a mutant that mimics non-phosphorylated AQP2 [41,42]. AQP2 mutants causing autosomal dominant NDI are expected to form heterotetramers with wt-AQP2, which are misrouted [52,56,58,63,64,65]. For both R254L and R254Q, formation of the hetero-tetramers with wt-AQP2 and the consequent retention in intracellular vesicles have been experimentally proven [41,42]. The ability of the R254L and R254Q mutants to form heterotetramers with wt-AQP2 monomers confirms a functional folding for their transmembrane domains.

### 2.5. Previously Described Structural Basis for Other AQP Mutants

#### 2.5.1. AQP2 (Q57P, N68S, A70D, V71M, G100R, T125M, T126M, A147T, V168M, P185A, R187C, R187H, E258K, P262L)

The structural basis for 12 AQP2 SAPs associated to NDI has been investigated by Frick et al. [21]. Five of them involve key functional residues: N68 (logo position 23 in Figure 2), A70, and P185 of the NPA region and R187 of the ar/R selectivity filter. Two other mutations, both from valine residues, V71M and V168M, were shown to cause pore narrowing [21]. These valines are largely conserved in aquaporins, as it can be seen from our pore-logo representation in Figure 2, where they occupy positions 21 and 26, respectively. G100R, located at a helix–helix interface of the AQP2 monomer, was hypothesised to disturb the correct folding of the protein. Other 2 mutations, T125M and T126M, are located in the extracellular loop C, close to an N-glycosylation site at N123. T125 is part of a canonical N-glycosylation consensus site, N123-X-T125, required for glycosylation at N123 [52]. Glycosylation at this site has been suggested to be important for protein sorting in the Golgi complex, as the N123Q mutant, lacking glycosylation, is retained in this organelle when expressed in renal cells [66]. In oocytes, however, expression of T125M and T126M leads to ER retention [48,52], whereas the glycosylation mutant N123Q does not [66]. Therefore Frick et al. suggested that it might not be the lack of glycosylation *per se* that causes ER retention but rather mutation-induced structural changes within loop C [21]. As loop C occupies a crucial position at the interface between two adjacent monomers, at the extracellular side of the protein, we assigned these mutations as tetramer assembly-affecting (see Table 2). Finally, the Q57P and A147T mutations were shown to disrupt the binding site of a divalent metal ion (Cd^2+^ in the crystal structure, presumably Ca^2+^ in vivo). This is reported in Table 2. However, we notice that the substitution of Q57, on Helix-2, with a proline could also affect the monomer folding, while the substitution of A147, at the interface between two adjacent monomers, with a threonine, would cause inter-monomer clashes, thus also possibly affecting the tetramer assembly.

As mentioned before, for the two mutants E258K and P262L a characterisation on a structural basis is not feasible, as the longest AQP2 structure available (PDB ID: 4OJ2) ends at residue V257. However, previous experimental studies have shown that repulsion between K258 and upstream arginines explains the mis-sorting of the AQP2 E258K mutant in NDI [67]. When co-expressed with wt-AQP2, E258K exhibits a dominant-negative effect, caused by impaired routing of wt-AQP2 to the plasma membrane because of hetero-tetramerization with the mutant [63].

Finally, P262L represents an exception because, despite being located on the AQP2 C-terminal tail, it causes recessive NDI. It is known that it is properly folded but causes intracellular vesicles retention, probably as a consequence of interfering with phosphorylation at S261 [44]. The P262L mutant interacts with wt-AQP2 but the resulting heterotetramers properly localise to the apical membrane, thus explaining the recessive character of such mutation [56].

#### 2.5.2. AQP5 (A38E, I45S, N123D, I177E, R188C)

All the 5 AQP5 SAPs associated with PPKB have been functionally and structurally characterized in Reference [25]. To easily visualise the localization of these mutations along the sequence, the human AQP5 monomer structure and sequence are shown in Figure S4.

Of these five mutations, I45S, I177F and R188C have been discussed as pore-affecting. R188 is the largely conserved arginine of the functional ar/R site (pore-logo position 30 in Figure 2), while I45 and I77 (pore-logo position 40) line the extracellular side of the water channel. We report in Figure S5 the pore radius along the channel coordinate and the channel profile of the I177F mutant, clearly showing how the substitution of an isoleucine into a larger phenylalanine leads to a significant pore narrowing at the extracellular side. We notice that the I45S mutation could also affect the packing between Helix-2 and Helices 1/5, as I45 in wt-AQP5 is involved in hydrophobic interactions with A32 on Helix-1, P40 and F49 on Helix-2 and I177 on Helix-5, while the mutated S45 cannot establish such interactions.

AQP5 mutations A38E and N123D are both located on the extra-membrane surface, in loop A and loop C, respectively, at the interface with another monomer. Mutation of A38 and N123 to charged amino acids has been hypothesized to affect the conformation of the loops and, consequently, the tertiary and especially the quaternary structure of the protein [25]. As we show in Figure S6, although the mutated residues are easily accommodated in the respective loops without introducing particular structural strain, they clearly change the electrostatic potential around the mutation site. This could be at the basis of the structural change hypothesized in Reference [25].

## 3. Materials and Methods

### 3.1. SAPs Collection

All the 34 AQP SAPs associated to genetic disorders were collected from the Uniprot/Swiss-Prot protein knowledgebase (humsavar.txt file, release 2017_09 of 27 September 2017; downloaded from www.uniprot.org). We selected this source as it is the most comprehensive. In OMIM [68] overall 20 SAPs are reported for AQPs (15 for AQP12 and 5 for AQP5), representing a subset of the 34 SAPs reported in HUMSAVAR.

### 3.2. Model Building

The homology model of hAQP8 was built with Modeller9v11 [69] using the HHpred interactive server (https://toolkit.tuebingen.mpg.de/#/hhpred) [70]. The structure of hAQP2 (PDB ID: 4NEF, resolution 2.75 Å, [21]) and hAQP4 (PDB ID: 3GD8, resolution 1.80 Å, [38]) were used as templates. Target-to-template sequence identities were 30% and 28%, respectively. The model quality was tested by ProQ, finding LG-score and MaxSub values of 3.969 and 0.297, respectively, that place it between correct and good quality models [71].

Models for hAQP2, hAQP5 and hAQP8 featuring the above mutations were built using the mutate_model module of the Modeller 9v11 program. This is an automated method for modelling point mutations in protein structures, which includes an optimisation procedure of the mutated residue in its environment, beginning with a conjugate gradients minimisation, continuing with molecular dynamics with simulated annealing and finishing again by conjugate gradients. The used force field is CHARM-22, for details see Reference [72]. Models of the 12 trans-membrane hAQP2 mutants were built starting from the X-ray structure of hAQP2 at better resolution (PDB ID: 4NEF, resolution 2.75 Å). As this structure ends at residue L240, thus lacking the C-terminal tail, models for the 2 R254L, R254Q mutants were instead built starting from a hAQP2 X-ray structure featuring worse resolution but ending at residue V257 (PDB ID: 4OJ2, resolution 3.05 Å, see Table 1). A superimposition of the two hAQP2 structures is shown in Figure S7. Models for the 5 hAQP5 mutants were built starting from the X-ray structure of hAQP5 (PDB ID: 3D9S, resolution 2.00 Å, [39]) and that for the hAQP8 mutant starting from the homology model we obtained for hAQP8.

### 3.3. Model Analysis

The molecular models were analysed and visually inspected using Pymol [73] and UCSF Chimera [74]. The COCOMAPS web server [75] was used to analyse inter-residue interactions between the trans-membrane helices within or between the monomers.

To visualise and to calculate pore diameters the HOLE program was used [49]. Electrostatic potentials were calculated by solving the Poisson-Boltzman (PB) equation using the PBEQ Solver package (http://www.charmm-gui.org/?doc=input/pbeqsolver, [76]). Calculations were performed by using the CHARMM force field, and the following default parameters: dielectric constant for the solute interior (EpsP) of 1.0; dielectric constant for the reference environment (EpsR) of 1.0; solvent dielectric constant (EpsW) of 80.0; coarse grid spacing (Dcel_c) of 1.5 Å and finer grid spacing (Dcel_f) of 1.0 Å.

### 3.4. Pore-Logos

Human AQPs sequences have been collected from the Uniprot database [77] and aligned by Clustal W [78]. Human AQPs pore-logos have been derived from 51 sequence alignment positions of the aquaporin family, as detailed in [46]. Sequences logos of the pore-lining residues have been obtained by the WebLogo3 web tool (http://weblogo.threeplusone.com/create.cgi) [79].

## 4. Conclusions

In this work, we addressed the structural basis of human AQP SAPs reported in Uniprot/Swiss-prot as associated with genetic disorders. We provided a detailed in silico structural investigation for all the mutants not structurally characterised so far, and integrated results of such analyses with those already reported for the remaining mutants. Besides providing a comprehensive overview, our study reveals a clear correlation between the type of mutation-induced structure defect and the experimentally observed phenotypes for mutations located on the AQP trans-membrane domains. Specifically, mutations affecting the monomer folding by altering the intra-monomer helices packing (the majority among those analysed here) cause the most severe phenotypes. These mutants are indeed non-functional (no water passage under osmotic gradient observed), since unfolded and therefore ER retained. The functionality of mutants whose pore signature motifs—NPA boxes and the ar/R selectivity filter—are affected is also compromised. However, mutations affecting other features of the pore, such as its dimension and composition, or the tetramer assembly are associated with milder phenotypes, with resulting mutants partially retaining their water channel functionality. This study may thus represent a reference frame for the interpretation and prediction, on a structural basis, of defective phenotypes of other AQP pathogenic mutants.

Nowadays, the therapeutic approach for genetic diseases based on the rescue of defective phenotypes of pathogenic mutants by pharmacological chaperones is considered one of the most promising strategies, particularly for NDI [80]. Cell and mouse models indeed supported the evidence that some forms of NDI caused by AQP2 mutations can be treated by pharmacological chaperons. In particular, for a few mutants retaining at least partially the water channel functionality, it has been shown that molecular chaperones could cause a redistribution of the mutants from the ER to the plasma membrane and endosomes and, in an adult mouse model, deal to partial restoration of urinary concentration function [18,51,81]. In this scenario, we remark the relevance of assigning and possibly predicting on a structural basis the severity of the defective phenotypes of AQP mutants associated to genetic diseases.

## Figures and Tables

**Table 1:** Experimental 3D structures available for human AQPs from the wwPDB (worldwide Protein Data Bank, last update on January 2018).

hAQP	Ress	PDB ID	Mutation	Asymmetric Unit	Pore ^1^	Method ^2^	Resolution (Å)	Deposition
AQP1	8-233	1FQY [34]	--	monomer	--	EC	3.80	2000
	9-233	1H6I [35]	--	monomer	--	EC	3.80	2001
	9-232	1IH5 [36]	--	monomer	--	EC	3.70	2001
	3-235	4CSK [37]	--	monomer	--	XRD	3.28	2014
AQP2	2-240	4NEF [21]	--	tetramer	--	XRD	2.75	2013
	5-257	4OJ2 ^3^	S256A	monomer	water	XRD	3.05	2014
AQP4	32-254	3GD8 [38]	--	monomer	water	XRD	1.80	2009
AQP5	1-245	3D9S [39]	--	tetramer	water	XRD	2.00	2008
	2-245	5C5X [40]	S156E	octamer	water	XRD	2.60	2015
	2-244	5DYE [40]	S156E	tetramer	--	XRD	3.50	2015

^1^ Molecules present in the pore. ^2^ EC stands for Electron Crystallography and XRD for X-ray Diffraction. ^3^ Not published.

**Table 2:** Aquaporin SAPs associated to genetic diseases reported in Uniprot/Swiss-Protprotein knowledgebase (HUMSAVAR, updated to September 2017). Mutations whose structural basis is analysed here for the first time are highlighted. The hosting protein, specific SAP, associated disease (NDI: Nephrogenic Diabetes Insipidus, PPKB: PalmoPlantar Keratoderma of Bothnian type, colorectal tumor), structural location and hypothesized structural defect are reported for each mutant. When the mutation affects a residue of the NPA motifs or the ar/R site, it is indicated in parentheses with the mutated residue underlined.

hAQP	Mutation	Disease	Location	Hypothesized Structural Defect
AQP2	L22V	NDI	H1	tetramer assembly
AQP2	L28P	NDI	H1	monomer folding
AQP2	A47V	NDI	H2	monomer folding
AQP2	Q57P	NDI	H2	impaired metal binding [21]
AQP2	G64R	NDI	H2	pore features
AQP2	N68S	NDI	HB	pore features (NPA) [21]
AQP2	A70D	NDI	HB	pore features (NPA) [21]
AQP2	V71M	NDI	HB	pore features [21]
AQP2	G100R	NDI	H3	monomer folding [21]
AQP2	G100V	NDI	H3	monomer folding
AQP2	T108M	NDI	H3	monomer folding
AQP2	T125M	NDI	loop C	tetramer assembly [21]
AQP2	T126M	NDI	H4	tetramer assembly [21]
AQP2	A147T	NDI	H4	impaired metal binding [21]
AQP2	V168M	NDI	H5	pore features [21]
AQP2	G175R	NDI	H5	monomer folding
AQP2	G180S	NDI	H5	pore features
AQP2	C181W	NDI	loop E	monomer folding
AQP2	P185A	NDI	HE	pore features (NPA) [21]
AQP2	R187C	NDI	HE	pore features (ar/R) [21]
AQP2	R187H	NDI	HE	pore features (ar/R) [21]
AQP2	A190T	NDI	HE	monomer folding
AQP2	W202C	NDI	H6	monomer folding
AQP2	S216P	NDI	H6	monomer folding
AQP2	R254L	NDI	C-ter	signal loss [41]
AQP2	R254Q	NDI	C-ter	signal loss [42]
AQP2	E258K	NDI	C-ter	signal loss [43]
AQP2	P262L	NDI	C-ter	signal loss [44]
AQP5	A38E	PPKB	loop A	tetramer assembly [25]
AQP5	I45S	PPKB	H2	pore features [25]
AQP5	N123D	PPKB	loop C	tetramer assembly [25]
AQP5	I177F	PPKB	H5	pore features [25]
AQP5	R188C	PPKB	HE	pore features [25]
AQP8	I229M	colorectal tumor	H6	monomer folding

## Supplementary Materials

Supplementary materials can be found at http://www.mdpi.com/1422-0067/19/6/1577/s1.

## Abbreviations

AQP	Aquaporin
CHO	Chinese Hamster Ovary
ER	Endoplasmic Reticulum
NDI	Nephrogenic Diabetes Insipidus
PPKB	PalmoPlantar Keratoderma of Bothnian type
SAP	Single Amino acid Polymorphism
