# Supplementary material for: Structural Basis for Mutations of Human Aquaporins Associated to Genetic Diseases
Source: Int J Mol Sci. 2018 May 25;19(6):1577. doi: 10.3390/ijms19061577 (PMC6032259; doi:10.3390/ijms19061577)
Supplement: Supplementary file 1 [file ijms-19-01577-s001.pdf]

Structural basis for mutations of human aquaporins associated to genetic diseases

Luisa Calvanese[1], Gabriella D'Auria[1,2,3], Anna Vangone[4], Lucia Falcigno[1,2,3], Romina Oliva[5,*]

[1]*CIRPeB, University of Naples Federico II, Napoli (Italy);* [2]*Department of Pharmacy, University of Naples FedericoII, Napoli (Italy) ;* [3]*Institute of Biostructures and Bioimaging, CNR, Napoli (Italy);*[4]*Bijvoet Center for Biomolecular Research, Faculty of Science - Chemistry, Utrecht University, Utrecht (The Netherlands);*[5]*Department of Sciences and Technologies, University Parthenope of Naples, Napoli.*

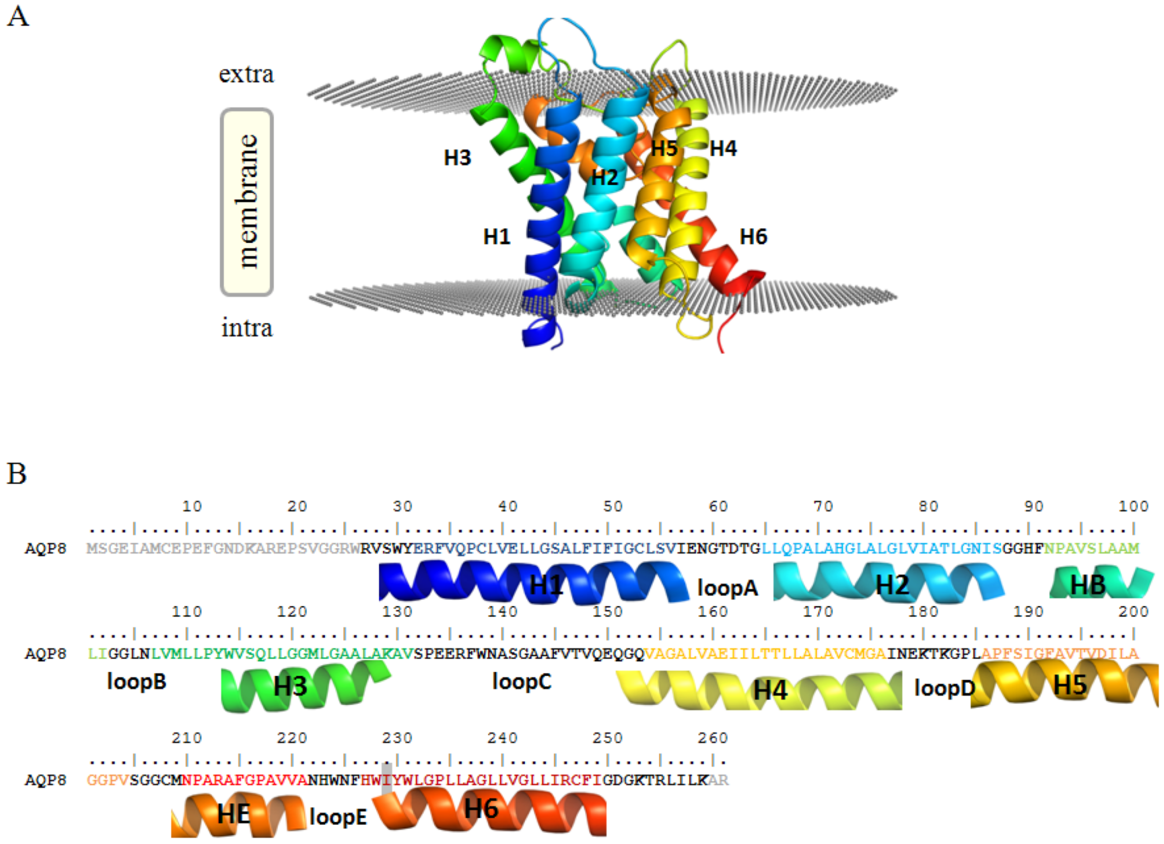

Figure S1. Human AQP8 monomer. A) Comparative model and **B)** sequence. Each helix is differently colored and common labels are shown. The mutated residue here examined is highlighted in the sequence (Fig. S1B). The not modeled N- and C-terminal residues are shown in light gray.

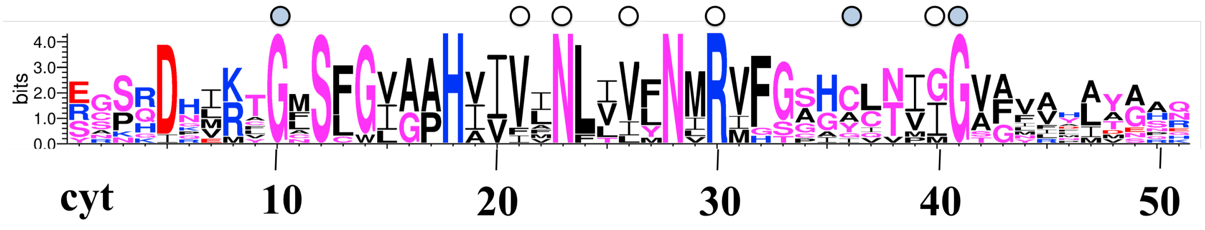

Figure S2. Pore logo representation of human aquaporins. Sequence logo of the pore-lining residues for all human canonical and glycerol-permeable AQPs. The logo numbers correspond to the structural position of the residues along the channel axis and not to their sequence position. Each pore is orientated from the intracellular (left) to the extracellular (right) side. Acidic residues are colored in red, basic residues in blue, hydrophobic residues in black and polar residues in purple. The pore positions where mutations occur, which are hypothesized to affect the pore features herein or in previous studies are pointed out by a filled or an empty circle, respectively.

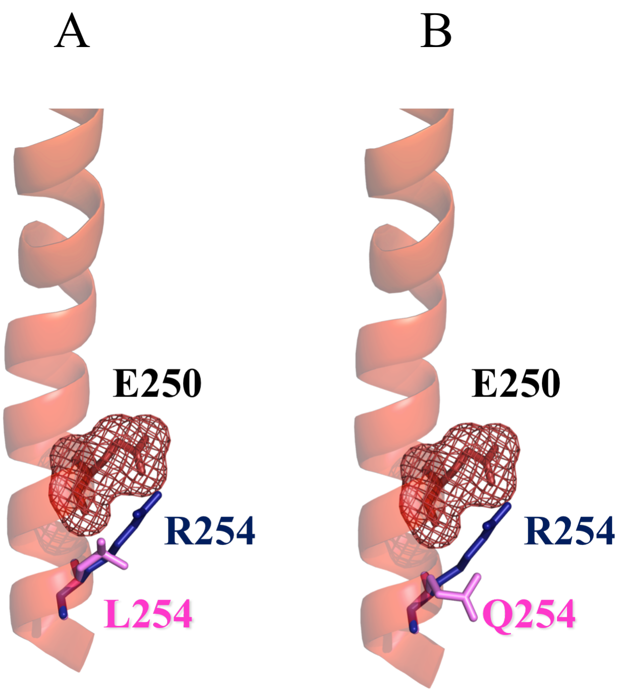

Figure S3. AQP2 R254L and R254Q mutants. Cartoon representation of the C-terminal tail of the: A) AQP2 R254L mutant and B) R254Q mutant, both superimposed on the wt-AQP2. Residues at position 254 are shown as sticks, and colored blue (wild-type) and violet (mutants). The native R254 residue establishes a salt-bridge interaction with E250, shown in red stick and mesh surface, which is missing when R254 is mutated to L or Q.

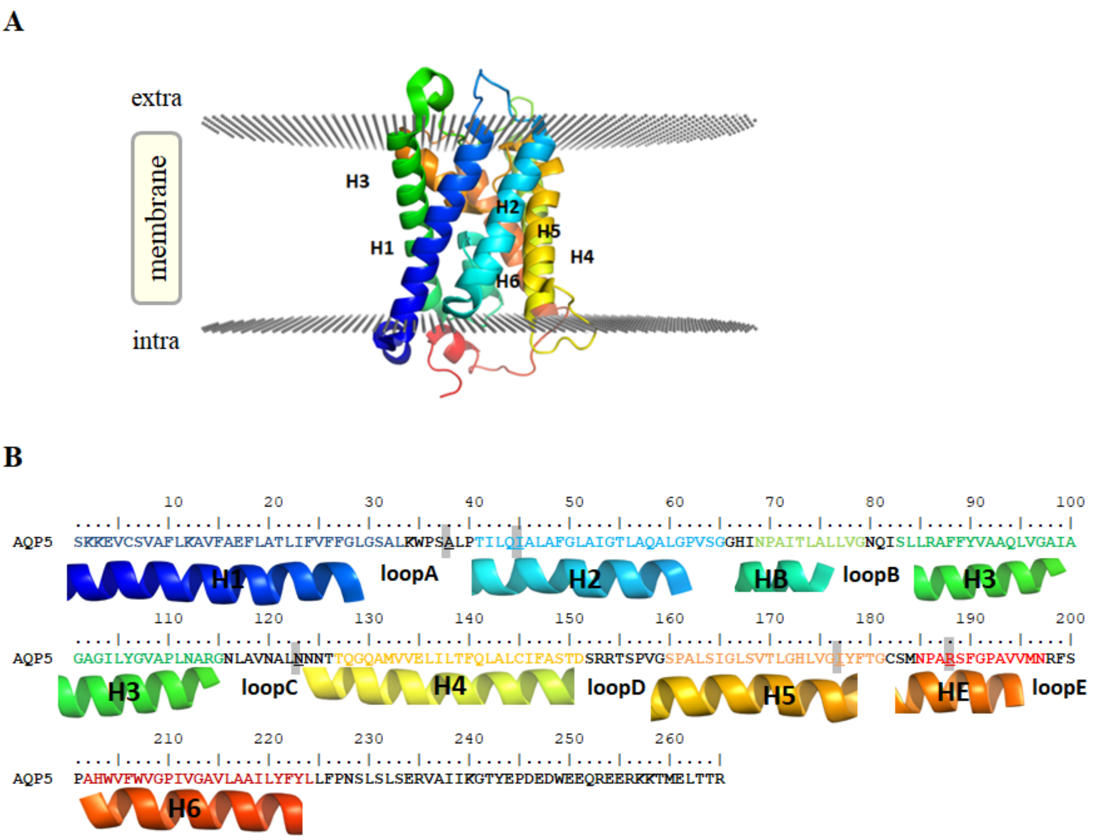

Figure S4. Human AQP5 A) structure (PDB ID: 3D9S) and B) sequence. Each helix is differently colored and common labels are reported. Residues affected by PPKB-associated mutations are highlighted in gray.

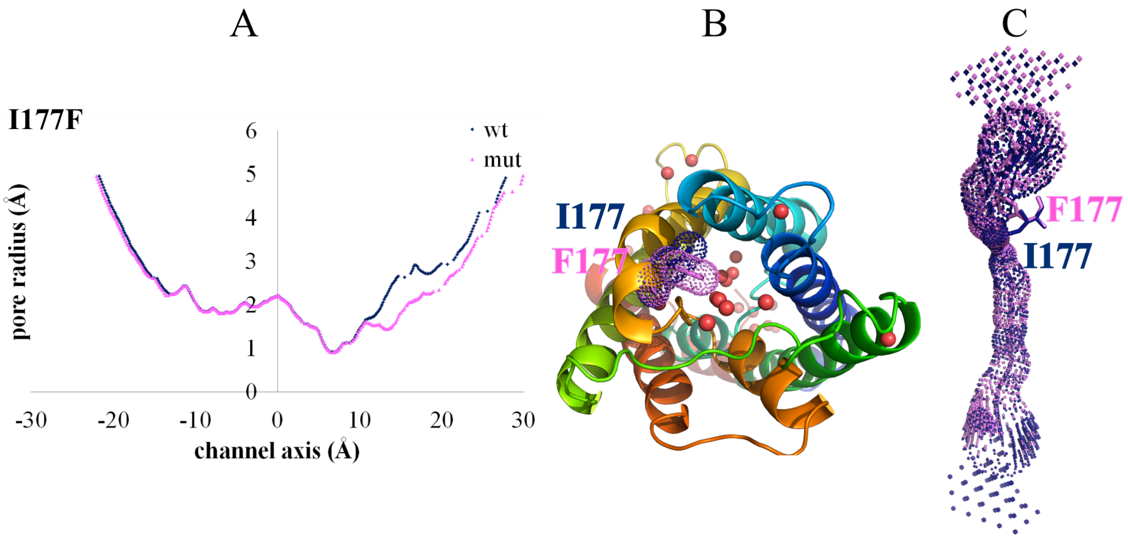

Figure S5. Pore features of the AQP5 I177F mutant. A) Pore radius vs. channel coordinates for wt-AQP5 (blue) and I177F (violet). B) Cartoon representation with the mutated residue in violet sticks and water molecules represented as small red spheres. C) Pore dimensions have been calculated by the program HOLE for wt-AQP5 (blue spheres) and I177F (violet spheres).

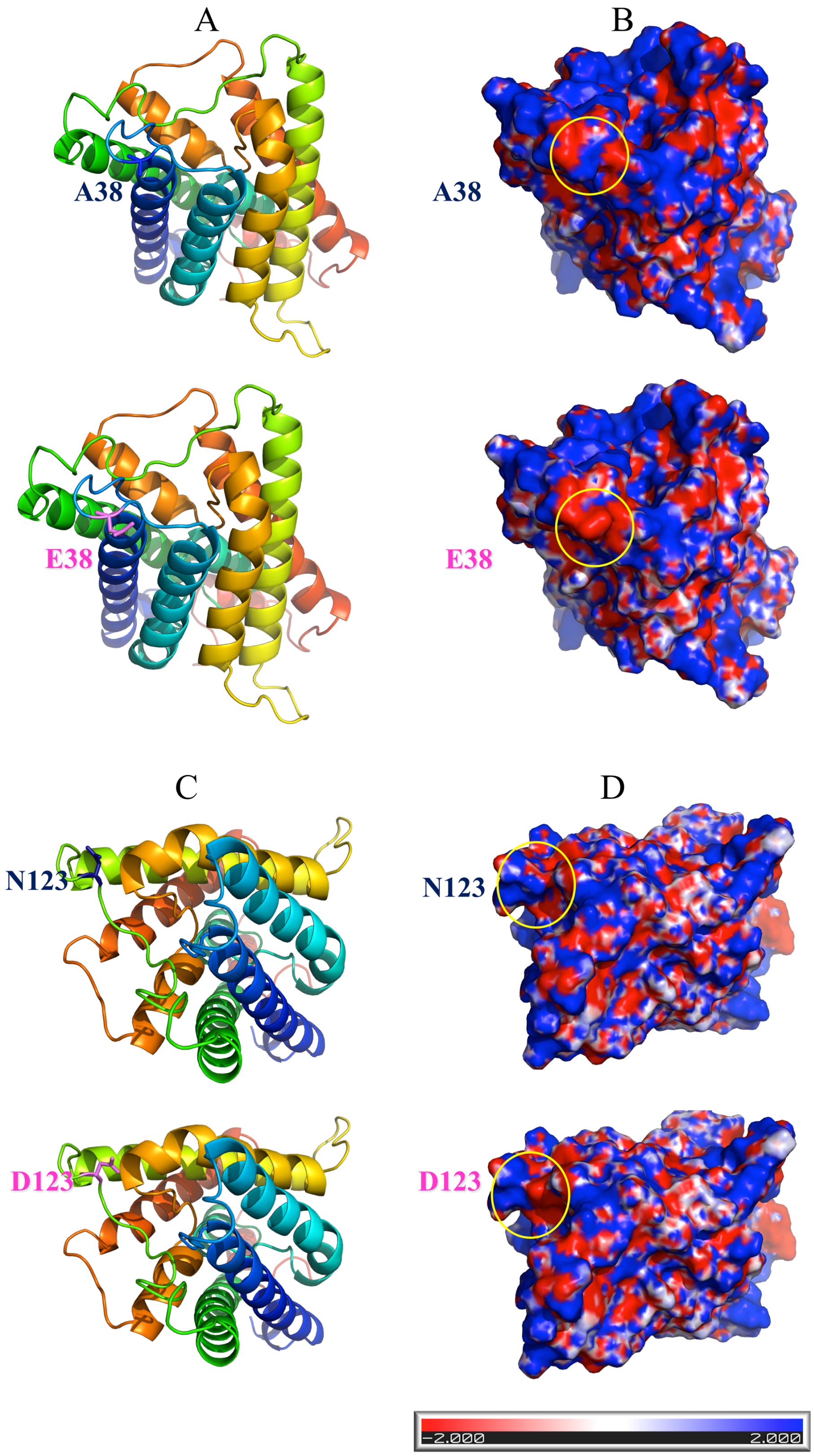

Figure S6. AQP5 A38E and N123D mutants. A) Cartoon and **B**) surface representation colored by electrostatic potential values of human wt-AQP5 compared to the A38E and N123D mutants.

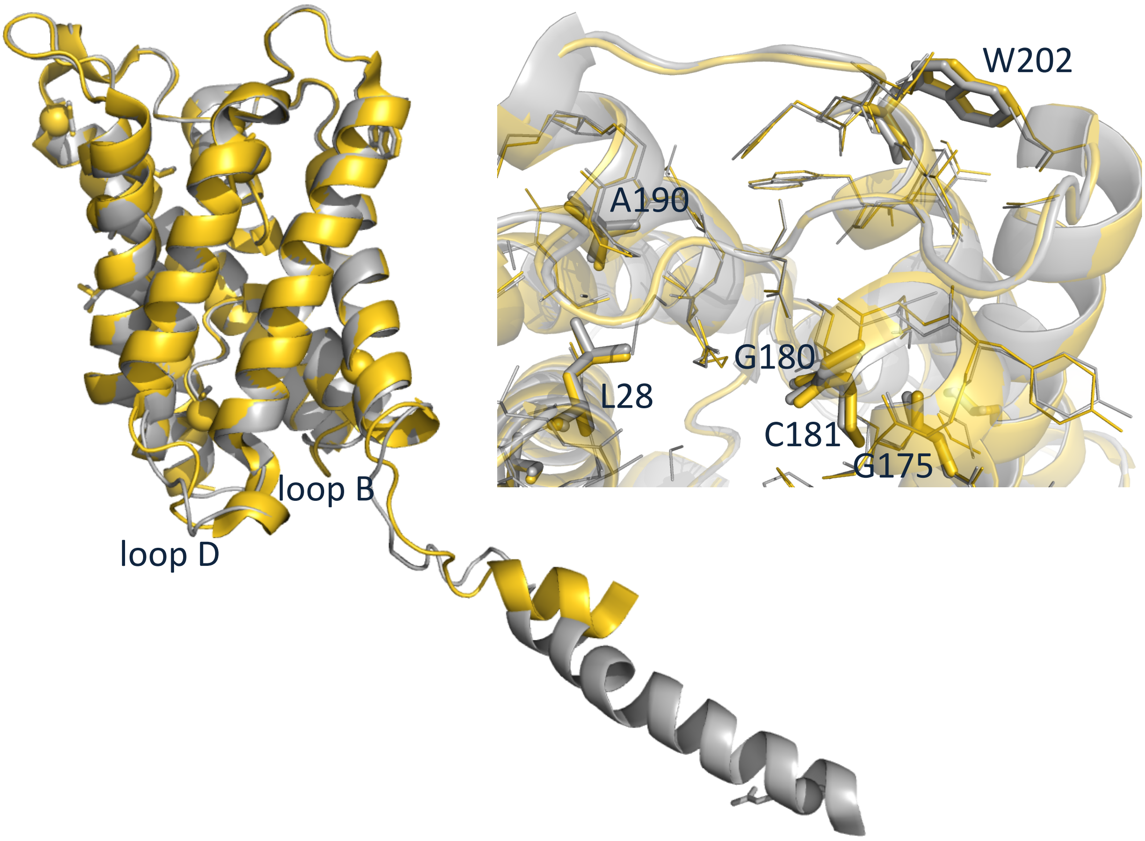

Figure S7. Best superimposition between the two hAQP2 structures used for modeling the hAQP2 SAPs. The 4NEF structure (resolution 2.75 Å) is colored in gold and the 4OJ2 structure (resolution 3.05 Å) is colored in silver. **Left:** cartoon representation with loops B and D, experiencing small conformational changes, and the C-terminal tail, truncated and exhibiting a different orientation in 4NEF, on display. Mutated residues are shown as sticks and with a sphere centered on their Cα on the respective structures. **Right:** focus on a hAQP2 inter-membrane region including six mutated residues (in sticks) and their neighbor residues, within 5 Å (in lines). The two monomers can be superimposed from the first residue of Helix-1 to the last residue of Helix-6 (for a total of 216 residues) with an rmsd value of 0.74 Å on the backbone atoms and of 1.4 Å on all the heavy atoms. Upon excluding from the superimposition loops B and D, the rmsd values decrease to 0.52 Å and 1.1 Å, respectively.
